# Cyclosporine Treatment Reduces Oxygen Free Radical Generation and Oxidative Stress in the Brain of Hypoxia-Reoxygenated Newborn Piglets

**DOI:** 10.1371/journal.pone.0040471

**Published:** 2012-07-09

**Authors:** Richdeep S. Gill, Tze-Fun Lee, Jiang-Qin Liu, Hetal Chaudhary, Dion R. Brocks, David L. Bigam, Po-Yin Cheung

**Affiliations:** 1 Department of Surgery, University of Alberta, Edmonton, Alberta, Canada; 2 Department of Pediatrics, University of Alberta, Edmonton, Alberta, Canada; 3 Faculty of Pharmacy and Pharmaceutical Sciences, University of Alberta, Edmonton, Alberta, Canada; Massachusetts General Hospital/Harvard Medical School, United States of America

## Abstract

Oxygen free radicals have been implicated in the pathogenesis of hypoxic-ischemic encephalopathy. It has previously been shown in traumatic brain injury animal models that treatment with cyclosporine reduces brain injury. However, the potential neuroprotective effect of cyclosporine in asphyxiated neonates has yet to be fully studied. Using an acute newborn swine model of hypoxia-reoxygenation, we evaluated the effects of cyclosporine on the brain, focusing on hydrogen peroxide (H_2_O_2_) production and markers of oxidative stress. Piglets (1–4 d, 1.4–2.5 kg) were block-randomized into three hypoxia-reoxygenation experimental groups (2 h hypoxia followed by 4 h reoxygenation)(n = 8/group). At 5 min after reoxygenation, piglets were given either i.v. saline (placebo, controls) or cyclosporine (2.5 or 10 mg/kg i.v. bolus) in a blinded-randomized fashion. An additional sham-operated group (n = 4) underwent no hypoxia-reoxygenation. Systemic hemodynamics, carotid arterial blood flow (transit-time ultrasonic probe), cerebral cortical H_2_O_2_ production (electrochemical sensor), cerebral tissue glutathione (ELISA) and cytosolic cytochrome-c (western blot) levels were examined. Hypoxic piglets had cardiogenic shock (cardiac output 40–48% of baseline), hypotension (mean arterial pressure 27–31 mmHg) and acidosis (pH 7.04) at the end of 2 h of hypoxia. Post-resuscitation cyclosporine treatment, particularly the higher dose (10 mg/kg), significantly attenuated the increase in cortical H_2_O_2_ concentration during reoxygenation, and was associated with lower cerebral oxidized glutathione levels. Furthermore, cyclosporine treatment significantly attenuated the increase in cortical cytochrome-c and lactate levels. Carotid blood arterial flow was similar among groups during reoxygenation. Conclusively, post-resuscitation administration of cyclosporine significantly attenuates H_2_O_2_ production and minimizes oxidative stress in newborn piglets following hypoxia-reoxygenation.

## Introduction

Asphyxia contributes to over 1 million neonatal deaths per year worldwide, with hypoxic-ischemic encephalopathy (HIE) being the most common morbidity in survivors [1]. In the US, HIE is estimated to occur in 1 to 4 cases per 1000 live births [2]. Globally, approximately 10% to 60% of these neonates with HIE will die, with more than 25% of survivors developing long-term neurodevelopmental complications [3], [4]. Specifically, HIE may result in disruption of long-term learning and memory in the survivors. In addition, these asphyxiated neonates may suffer from seizures, feeding difficulties and neuromotor impairment [3], [4].

Although the exact mechanisms have not been fully elucidated, Rodrigo et al proposed that oxygen free radicals played an important role in reperfusion/reoxygenation injury following asphyxia [5]. Excess production of reactive oxygen species (ROS) such as superoxide anion, hydroxyl radical, hydrogen peroxide (H_2_O_2_) and nitric oxide has been reported during ischemia-reperfusion or hypoxia-reoxygenation (H–R). These ROS and their metabolites cause cellular damage and cell death by oxidizing proteins, inducing lipid peroxidation and damaging DNA. There is evidence that mitochondrial dysfunction plays an essential role in both apoptotic and necrotic cellular death in HIE [6], [7]. Mitochondrial damage secondary to oxidative stress during reperfusion/reoxygenation has been proposed to be associated with opening to the mitochondrial permeability transition pore (MPTP), which uncouples oxidative phosphorylation and leads to mitochondrial swelling. Swelling may lead to rupture of the outer mitochondrial membrane and release of apoptotic signaling molecules such as cytochrome-c [6], [7], [8].

Cyclosporine A has been shown *in vivo* to reduce swelling of isolated brain mitochondria [9]. Furthermore, experimental studies show that cyclosporine A inhibits opening of the MPTP by binding to cyclophilin-D [10]. This raises the possibility that maintaining cell integrity by cyclosporine may have certain beneficial effects in neuroprotection. In animal models of traumatic brain injury, cyclosporine treatment has been shown to reduce axonal injury [11] and attenuate lipid peroxidation [12]. Only a few studies have been carried out to examine the effectiveness of cyclosporine treatment in neuroprotection of the immature newborn brain. A significant improvement against ischemic/hypoxic-induced brain injury following cyclosporine treatment has been reported in both fetal and newborn rats using models of in utero ischemia and carotid/cerebral artery ligation, respectively [8], [13], [14]. In contrast, Puka-Sundvall et al. reported that cyclosporine did not provide neuroprotection after hypoxia-ischemia in newborn rats [15]. Despite these controversial observations, the neuroprotective effects of cyclosporine have *not* been assessed in large-sized newborn animals that underwent global H-R as that in the clinical scenario. Our objective was to determine if post-resuscitation cyclosporine treatment would attenuate cerebral ROS (H_2_O_2_) production and oxidative stress-related injury in newborn piglets during asphyxia-reoxygenation. We hypothesize that cyclosporine treatment during resuscitation will reduce H_2_O_2_ generation and oxidative stress in the brains of asphyxiated newborn piglets.

## Methods

All experiments were conducted in accordance with the guidelines and approval of the Animal Care and Use Committee (Health Science), University of Alberta. Twenty-eight newborn mixed breed piglets, 1 to 4 days of age, weighing 1.4 to 2.5 kg were obtained on the day of experimentation from the Swine Research Technology Center (University of Alberta).

### Animal Preparation

Anesthesia was induced initially with isofluorane at 5% and maintained at 2% to 3%. Inhalational anesthesia with isofluorane was discontinued once mechanical ventilation was commenced. Subsequently anesthesia was maintained with intravenous fentanyl 5–20 µg/kg/h and midazolam 0.2–1.0 mg/kg/h. and pancuronium 0.05–0.1 mg/kg/h. Additional doses of fentanyl (10 µg/kg) and acepromazine (0.25 mg/kg) were also given intravenously as needed. Fractional inspired oxygen concentrations (FiO_2_) were continuously measured and maintained at 0.21–0.25 to keep arterial oxygen saturations between 90% and 100%. Oxygen saturation was measured by pulse oximeter (Nellcor, Hayward, California), whereas heart rate and blood pressure were measured by a Hewlett Packard 78833B monitor (Hewlett Packard Co, Palo Alto, California). IV fluids consisting of 5% dextrose in water at 10 ml/kg/h and 0.9% NaCl at 2 ml/kg/h were used to maintain glucose levels and hydration. Piglet body temperature was maintained at 38.5–39.5°C using overhead warmer and a heating pad.

A 5-French Argyle double-lumen catheter was inserted into the femoral vein, up to the level of the right atrium for administration of fluids and medications. A 5-French Argyle single-lumen catheter was inserted into the femoral artery to the distal aorta and attached to a pressure transducer for continuous systemic measurement of arterial pressure to determine mean arterial pressure (MAP). Endotracheal intubation via a tracheostomy was performed, and pressure-controlled ventilation (Sechrist infant ventilator model IV-100; Sechrist Industries, Anaheim, CA) was commenced at a respiratory rate of 16–20 breaths/min and pressure of 19/4 cm H_2_O. Meanwhile, an ultrasound flow probe (2RB, Transonic System Inc., Ithaca, NY) was encircled around the right common carotid artery for continuous measurement of carotid arterial blood flow (CABF). Other studies have suggested a good correlation between the common carotid and cerebral blood flows [16]. Transonic flow probes and pressure transducer outputs were digitized and recorded by a converter board in a computer equipped with custom Asyst programming software (Data Translation, Ontario, Canada).

The piglet was then placed in the prone position with the head mounted in a stereotaxic holder. Using bregma as the reference point, a stainless steel guide cannula (19-gauge) was implanted in the right frontoparietal cortex using the following co-ordinates: AP = 6.5 L = 4 H = 6 mm. The co-ordinates for the cerebral cortex were based on an atlas constructed with several pilot studies. This cerebral region was chosen because significant histological and biochemical injury was observed in previous studies [17], [18], [19]. The guide cannula was fixed on the skull with dental cement.

The piglets were allowed to recover from surgical instrumentation until baseline hemodynamic measures were stable. Ventilator rate was adjusted to maintain the P_a_CO_2_ 35–45 mmHg as determined by periodic arterial blood gas analysis during experimentation. Heart rate, MAP and CABF were continuously monitored and recorded throughout the experiment.

### Experimental Protocol

The piglets were block-randomized to 3 groups (n = 8 per group) that underwent H-R. A fourth sham-operated group of piglets (n = 4) underwent complete instrumentation without H-R.

In the 3 H-R groups, hypoxemia was induced via normocapnic alveolar hypoxia. These piglets were ventilated with a FiO_2_ of 0.10–0.15 by increasing the inhaled concentration of nitrogen gas relative to oxygen for 2 h, aiming for arterial oxygen saturations of 30–40%. It has been shown in previous studies that this degree of hypoxemia in the newborn piglet model will produce clinical asphyxia with severe metabolic acidosis and systemic hypotension [20], [21]. This was followed by reoxygenation with 100% oxygen for 0.5 h and then 21% oxygen for 3.5 h. At 5 min of reoxygenation, piglets received a blinded treatment either with cyclosporine as an intravenous bolus (2.5 or 10 mg/kg) or saline (H-R control). Cyclosporine A treatment was given at 5 min reoxygenation to simulate the clinical setting, in which intravenous access is obtained prior to administering resuscitative medications to the neonate.

Blinding was maintained by reconstituting all doses of cyclosporine and normal saline in a standard volume (5 ml) immediately before administration. The medication was given intravenously over 2 min. A laboratory technician, who was not involved in the experiment, prepared and administered the medications.

At the end of the study, piglets were euthanized with 100 mg/kg pentobarbital i.v. The whole brain was removed immediately and placed in 50 mL ice-cold 2-methylbutane for 10 min. After discarding 2-methylbutane, the brain was stored in −80°C for further biochemical analysis.

**Table 1:** Changes in mean arterial pressure (MAP), heart rate and arterial blood gases during hypoxia and reoxygenation.

	Normoxic Baseline	End of hypoxia	Reoxygenation		
			30 min	2 h	4 h
**MAP (mmHg)**					
H-R Control	68±5	28±3§	47±4§	42±3§	34±3§
CSA 2.5 mg/kg	78±3	31±2§	48±5§	41±3§	44±3§
CSA 10 mg/kg	72±2	31±2§	54±6§	40±2§	38±2§
Sham-operated	68±3	57±4*	50±3	49±2	45±2*
**Heart Rate (bpm)**					
H-R Control	181±12	213±8	211±9	225±9	216±11
CSA 2.5 mg/kg	167±7	210±9§	185±8	191±10	207±14
CSA 10 mg/kg	162±9	212±8§	199±7§	214±8	211±10§
Sham-operated	204±17	237±10	232±7	231±3	221±8
**pH**					
H-R Control	7.41±0.06	7.08±0.07§	7.19±0.09§	7.31±0.07	7.31±0.12
CSA 2.5 mg/kg	7.39±0.08	7.02±0.15§	7.14±0.16§	7.35±0.09	7.35±0.08
CSA 10 mg/kg	7.43±0.05	7.05±0.17§	7.16±0.17§	7.35±0.07	7.35±0.04
Sham-operated	7.39±0.02	7.39±0.02*	7.42±0.02*	7.39±0.02	7.41±0.04
**PaO_2_ (mmHg)**					
H-R Control	74±13	37±8§	348±96§	62±6	63±16
CSA 2.5 mg/kg	83±9	36±6§	383±86§	63±8	63±4
CSA 10 mg/kg	79±13	36±8§	413±36§	69±6	64±4
Sham-operated	67±5	68±3*	68±4*	77±18	76±17
**PCO_2_ (mmHg)**					
H-R Control	39±3	39±5	37±4	39±3	39±4
CSA 2.5 mg/kg	39±4	42±4	39±5	36±3	42±3
CSA 10 mg/kg	37±4	39±5	36±5	38±3	41±3
Sham-operated	37±2	41±3	40±6	40±3	37±3
**Base excess (mmol/L)**					
H-R Control	0.1±3	−17±3§	−13±3§	−6±3	−5±2
CSA 2.5 mg/kg	−1.0±3	−18±5§	−14±6§	−5±5	−2±5
CSA 10 mg/kg	0.5±2	−17±6§	−14±6§	−4±4	−2±2
Sham-operated	−2.0±2	−0.3±1*	1.5±3*	−0.9±3	−0.8±2
**Plasma Lactate (mmol/L)**					
H-R Control	4±1	14±4§	11±3§	7±2	6±3
CSA 2.5 mg/kg	4±1	15±4§	11±3§	5±2	3±2
CSA 10 mg/kg	4±1	14±5§	12±4§	6±3	3±1
Sham-operated	4±2	3±1*	2±1*	2±1	2±1

§ p<0.05 vs. normoxic baseline (2-way repeated measures ANOVA).

* p<0.05 vs. H-R controls (2-way repeated measures ANOVA).

### Hemodynamic Measurements

Hemodynamic recording for data analysis were carried out at specified time points: baseline (0 min), 60 and 120 min of hypoxia, 130 (10-min reoxygenation) and 150 min (30-min reoxygenation) reoxygenation with 1.0 FiO_2_, then at 180 (60-min reoxygenation), 240 (120-min reoxygenation), 300 (180-min reoxygenation) and 360 min (240-min reoxygenation) for reoxygenation with 0.21 FiO_2_. All recordings were calculated as a mean over 2 min of recording. At the specified time points, both arterial and venous blood samples were taken for blood gases, hemoglobin levels and co-oximetry.

**Figure 1:**
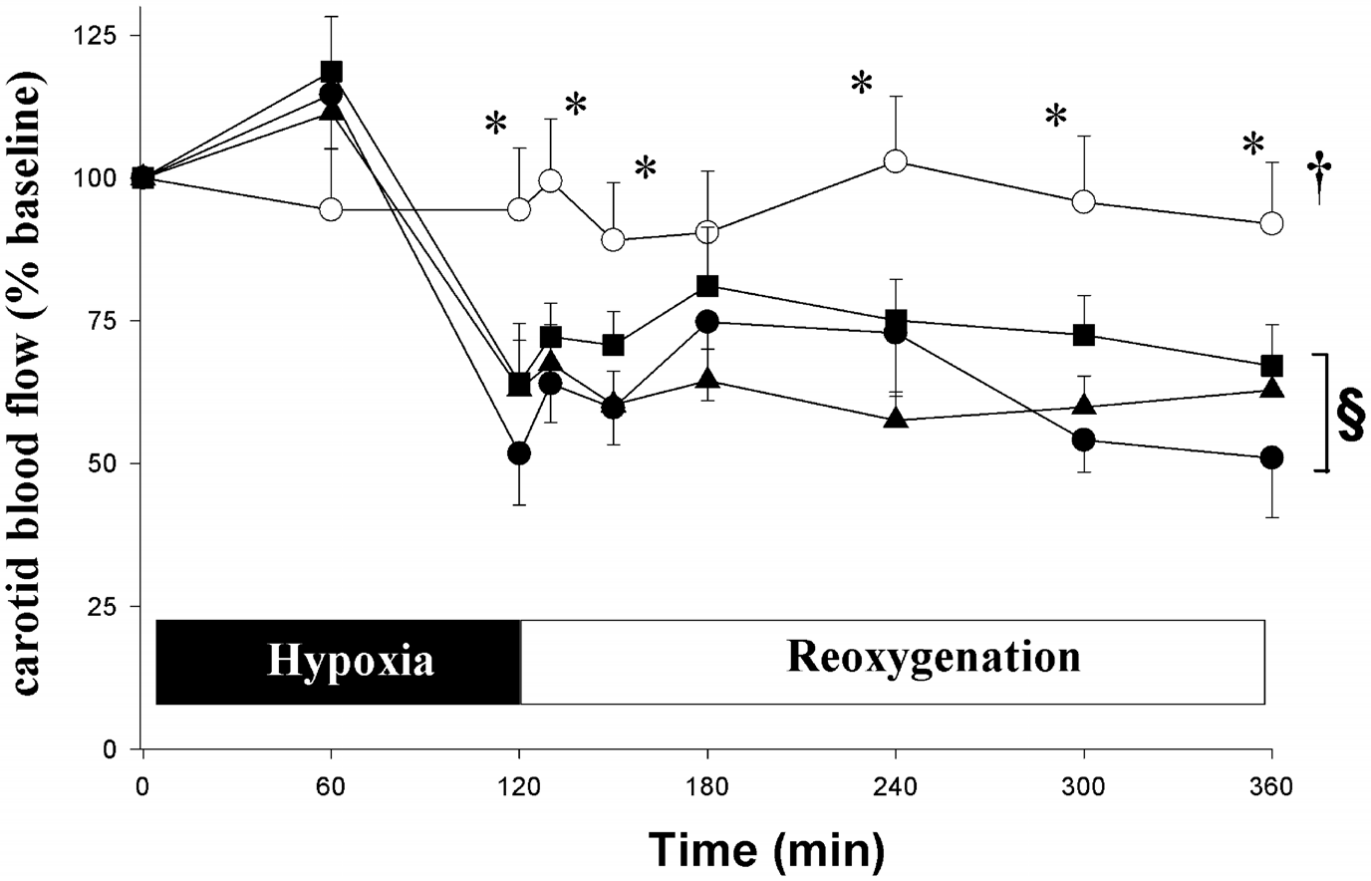
Temporal changes in carotid blood flow (CCAF) during 4 h of reoxygenation. Hypoxic piglets received with either saline (•, H–R control) or cyclosporine 2.5 (▴) or 10 mg/kg (▪) 5 min after reoxygenation. Sham-operated piglets underwent no hypoxia-reoxygenation (○). ^†^p<0.05 vs. H-R controls (2-way repeated measures ANOVA); *p<0.05 vs. H-R controls at simultaneous time point,^ §^p<0.05 vs. baseline (1-way ANOVA).

### Cerebral Cortical Hydrogen Peroxide Measurement

The change in cortical H_2_O_2_ during H-R was measured directly by electrochemical H_2_O_2_ sensor (HPO-100, World Precision Instruments Ltd.). After completion of surgery, the H_2_O_2_ sensor was inserted through the guide cannula into the cortex area. The sensors were connected to a computer-controlled data acquisition system (Apollo 4000, World Precision Instruments Ltd., Sarasota, FL). The signal outputs were recorded continuously throughout the experimental period. Immediately before and after each experiment, the H_2_O_2_ sensor was calibrated with 1 mM H_2_O_2_ in phosphate buffer (10 mM, pH 7.4). The phosphate buffer was pre-warmed to 38±1°C to adjust the temperature deviation. The mean value was used for converting the signal outputs. The relative change in cortical H_2_O_2_, expressed in µM, was calculated with reference to the normoxic baseline after stabilization.

### Determination of Cortical Glutathione and Lactate

A block of cortical tissue (5×5×5 mm^3^) from the left side of the cortex corresponding to the cannulation area of the right side was dissected. Part of the tissue was then homogenized with 5 µl/mg of 50 mM phosphate buffer containing 1 mM EDTA (pH 7.0) and stored at −80°C until biochemical analyses. The cortical level of GSH/GSSG was measured using commercially available assay kits (Cayman Chemical, #703002). Brain lactate was assayed by enzymatic spectrometric methods to measure the absorbance of NADH at 340 nm. The protein content was determined by bicinchoninic acid assay kit (Sigma).

**Figure 2:**
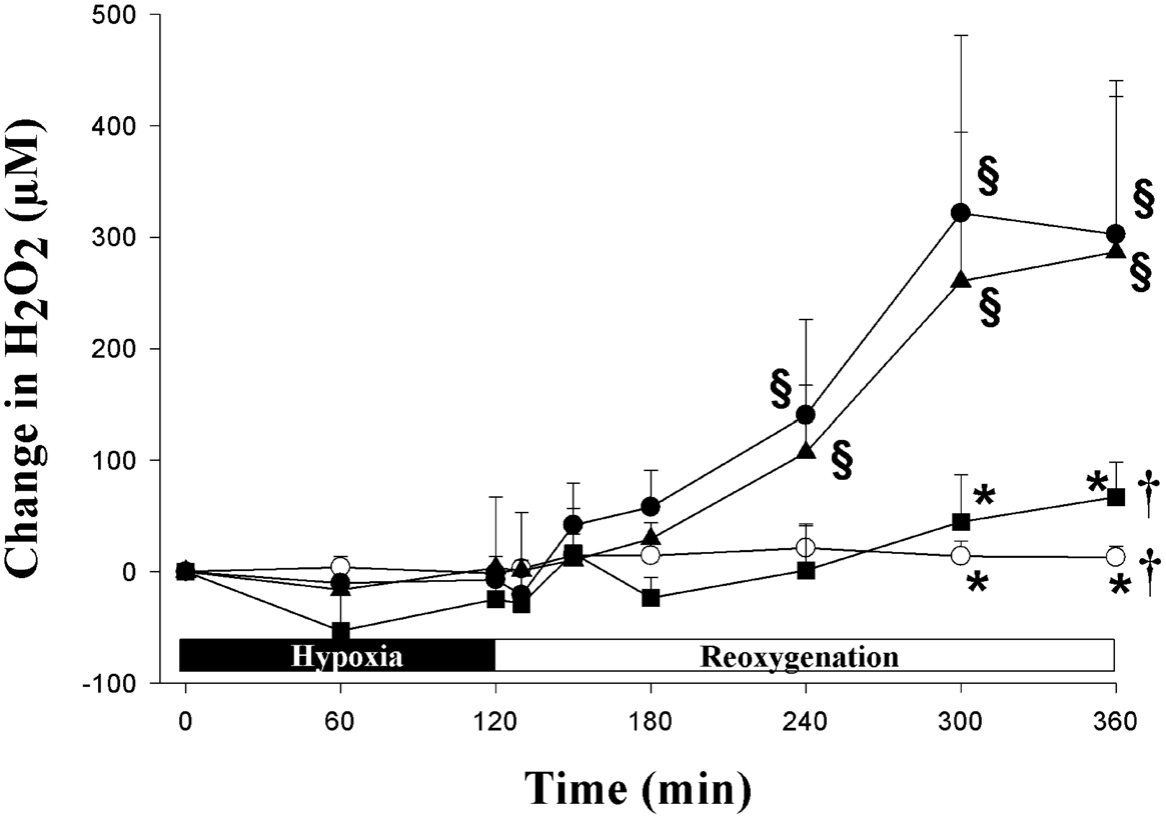
Temporal changes in cortical hydrogen peroxide (H_2_O_2_) concentration during 4 **h of reoxygenation.** Hypoxic piglets received with either saline (•, H-R control) or cyclosporine 2.5 (▴) or 10 mg/kg (▪) 5 min after reoxygenation. Sham-operated piglets underwent no hypoxia-reoxygenation (○). ^†^p<0.05 vs. H-R controls (2-way repeated measures ANOVA); *p<0.05 vs. H-R controls at simultaneous time point, ^§^p<0.05 vs. baseline (1-way ANOVA).

### Determination of Cytosol Cytochrome-c

Using polytron tissue grinder, part of the cortical tissue (∼100 mg) obtained as described above was homogenized with 250 µL cold homogenization buffer (20 mM Tris-HCl, pH 7.4, 50 mM NaCl, 50 mM NaF, 5 mM NaPP, 250 mM sucrose, and 1 mM dithiothreitol) supplemented with Complete protease inhibitor (Roche Diagnostic GmbH, Germany). The homogenates were centrifuged at 5,000 g for 10 min. Protein extract (50 µg) was mixed with loading buffer and denatured by boiling for 5 min before loading on a 12% sodium dodecyl sulfate-polyacrylamide gel electrophoresis gel. After electrophoresis, proteins were transferred to a 0.2-µm polyvinylidene difluoride membrane (Bio-Rad), and the latter were then blocked with 10% fat-free dry milk in Tween-Tris-buffer saline (TTBS) for 30 min at room temperature. After brief washing with TTBS, the membrane was incubated with a polyclonal antibody to cytochrome-c (dilution 1∶1,000; Biovision) overnight at 4°C cold room. After washing four times (5 min per wash) with TTBS to remove the unbound antibody, the membrane was then incubated in TTBS for 1 h at room temperature with horseradish peroxidase conjugated to goat anti-rabbit IgG (dilution 1∶1,000; EY Laboratories). Membranes were washed four times again with TTBS. After this, the membrane was re-incubated and blotted with ß-actin antibody in a similar fashion to determine the expression of ß-actin. Bound proteins were detected using chemiluminescence reagents (ECL plus; Amersham Biosciences) and visualized by exposing to x-ray film (Biomax MR, Kodak Photo Film). The film was developed by Kodak X-OMAT 1000A processor (Eastman Kodak Company). The x-ray films were scanned using PowerLook 1000 scanner (UMAX) and bands were analyzed using Quantity One 1-D analysis software (Bio-Rad). The integrated areas of bands were determined and expressed as a percentage of a standard sample ran on the same membrane. The amount of protein to be used for detection was normalized using the ß-actin as loading control.

### Determination of Cyclosporine A Levels in the Brain

For measurement of cyclosporine A in brain a validated LC/MS method was used with some modifications in extraction procedure [22]. To 500 mg of each tissue sample was 1.5 mL of distilled water (1∶3 w/w), which was then homogenized using a tissue grinder. Each homogenate was transferred to a clean glass tube, to which 40 µL of internal standard (amiodarone, 10 µg/mL), 500 µL of zinc sulfate (0.1 M) and 1.5 mL of acetonitrile were added. Samples were vortex mixed for 30 s and centrifuged for 10 min to precipitate the protein content. Supernatants were transferred in clean tubes and 200 µL of sodium hydroxide (1 M) was added. Drug and internal standard were extracted in 8 mL of ether: methanol (95∶5 v/v) by vortex mixing for 60 s and centrifuged for 10 min at 3000 g. The organic layer was transferred to new tubes and evaporated in vacuo. The dried residues were then reconstituted in 130 µL of methanol and 10 µL was injected in the LC/MS as previously described [22]. The mobile phase for brain was slightly modified to acetonitrile: methanol: 0.2% NH4OH (55∶20:25) pumped at an isocratic flow rate of 0.2 mL/min. The peak height ratio of cyclosporine to internal standard was used to quantify the cyclosporine A level in brain by comparing to a calibration curve. The calibration curve samples were prepared at the range of 10–1000 ng/mL concentration, with excellent linearity between concentration and height ratio (r^2^>0.99) and CV% being less than 15%.

### Statistical Analysis

All results are expressed as mean±SEM. Two-way repeated measures and 1-way analysis of variance and Krushal-Wallis test were used to study the differences between groups for parametric and non-parametric, respectively. *Post-hoc* testing with Fisher Least Significant Difference or Student-Newman-Keuls methods was performed for pairwise comparisons with the H-R control group as appropriate. Correlation between variables was studied by Pearson Moment or Spearman Rank Order test as appropriate. Statistical analyses were performed using SigmaPlot® (SPSS v11.0). Significance was set at p<0.05.

**Figure 3:**
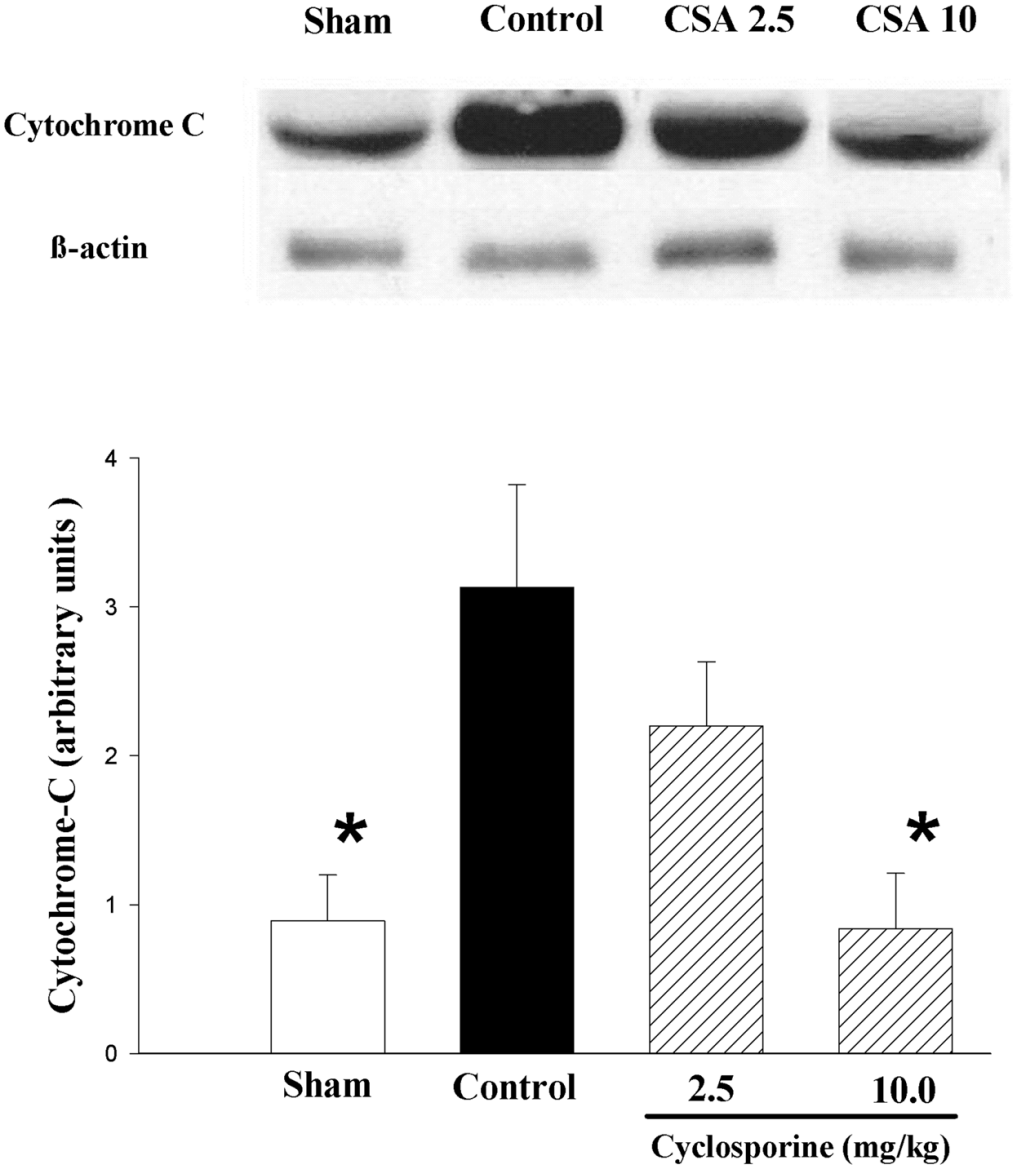
Representative western blots and levels of cytochrome-c (15 kDa) in brain cortical tissue of piglets after hypoxia-reoxygenation. Piglets received either saline (control) or cyclosporine 2.5 (CSA 2.5) or 10 mg/kg (CSA 10) 5 min after reoxygenation (n = 8 each). Sham piglets had no hypoxia and reoxygenation (n = 4). *p<0.05 vs. H-R controls (1-way ANOVA).

## Results

The piglets aged 2.3±0.2 day and weighed 1.9±0.04 kg with no significant differences among groups. Sham-operated animals were stable throughout the experimental period (Table 1).

### Effects of Cyclosporine on Physiological Parameters

MAP significantly decreased to 40% of the normoxic baseline value after 2 h of hypoxemia (p<0.05) (Table 1). After the immediate recovery upon reoxygenation, MAP of H-R controls deteriorated and remained lower than the normoxic baseline value throughout the reoxygenation period (p<0.05) (Table 1). The heart rate of hypoxic piglets was higher than the baseline value at the end of hypoxia and remained high throughout the reoxygenation period (Table 1). The temporal changes in MAP and heart rate during hypoxia and reoxygenation of cyclosporine-treated groups were not different from those observed in the H-R control group (Table 1).

**Table 2:** Effects of cyclosporine on cerebral cortical glutathione levels after hypoxic-reoxygenation (H-R).

	GSH(nmol/mg protein)	GSSG(nmol/mg protein)	GSSG:GSHRatio
H-R Control	229.3±20.4	27.2±1.5	0.12±0.01
CSA 2.5 mg/kg	272.7±35.8	24.6±3.4	0.09±0.01*
CSA 10 mg/kg	303.5±54.1	16.8±2.1*	0.06±0.01*
Sham-operated	330.1±47.3	18.2±3.5*	0.06±0.01*

* p<0.05 vs. H–R controls (one-way ANOVA).

The baseline value of CABF was 17.9±1 mL/min/kg (n = 28) with no difference among groups. The CABF of H-R controls was significantly lower than the baseline value at the end of hypoxia and remained low throughout reoxygenation (Fig. 1). Treating H-R piglets with cyclosporine did not significantly affect CABF (Fig. 1).

After exposure to hypoxia for 2 h, the arterial pH, PO_2_ and base excess levels of piglets decreased significantly below their baseline value (Table 1). After reoxygenation, all these parameters in blood gas recovered gradually towards the respective values at normoxic baseline. There were no significant differences in arterial blood gas between saline and cyclosporine-treated groups throughout hypoxia and reoxygenation (Table 1).

**Figure 4:**
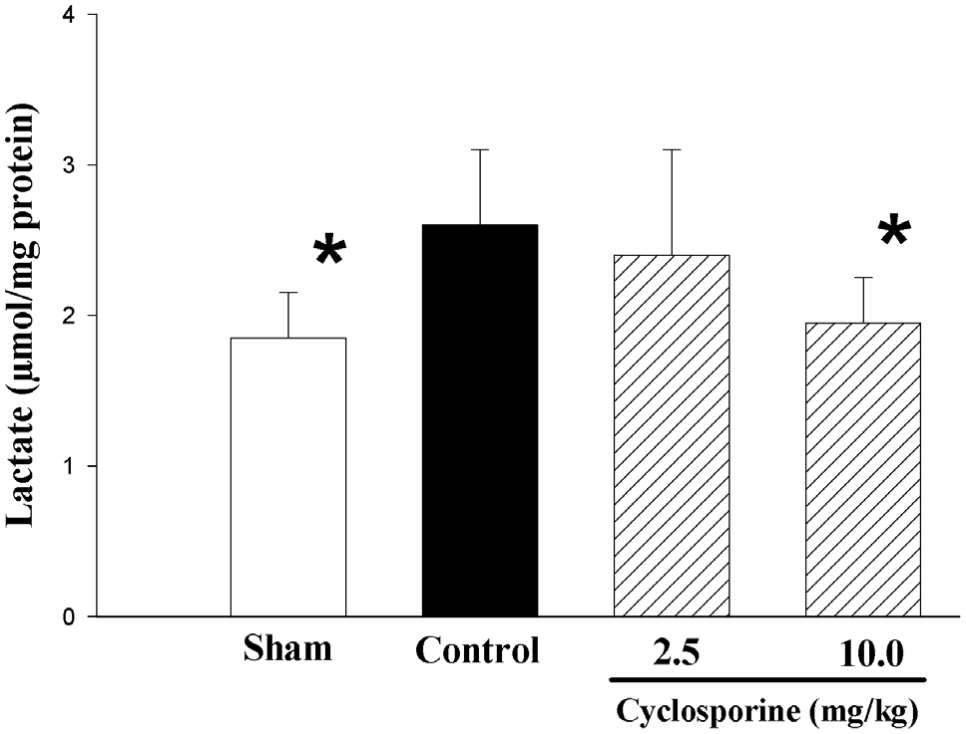
Changes in cerebral cortical lactate levels of piglets after hypoxia-reoxygenation. Piglets received either saline (control) or cyclosporine 2.5 (CSA 2.5) or 10 mg/kg (CSA 10) 5 min after reoxygenation (n = 8 each). Sham piglets had no hypoxia and reoxygenation (n = 4). *p<0.05 vs. H-R controls (1-way ANOVA).

### Effects of Cyclosporine on Cerebral Cortical Hydrogen Peroxide Production

Fig. 2 demonstrates the temporal changes in cortical H_2_O_2_ concentration during H-R. Cortical H_2_O_2_ concentrations in the H-R control and cyclosporine-treated groups maintained near baseline levels during hypoxia. After reoxygenation, the H_2_O_2_ concentration in H-R controls increased gradually within the first hour and then became significantly greater than the normoxic baseline for the remainder of experimental period (Fig. 2). Post-resuscitation administration of cyclosporine at 10 mg/kg, but not 2.5 mg/kg, abolished the increase in cortical H_2_O_2_ concentration during reoxygenation.

### Effects of Cyclosporine on Cortical Cytosol Cytochrome-c

The cytosol cytochrome-c level in the cerebral cortex of H-R controls was significantly higher than that of sham-operated group (Fig. 3). Treating the piglets with cyclosporine caused a dose-related reduction in cytochrome-c levels, with a significant difference observed between piglets treated with 10 mg/kg cyclosporine and H-R controls.

**Figure 5:**
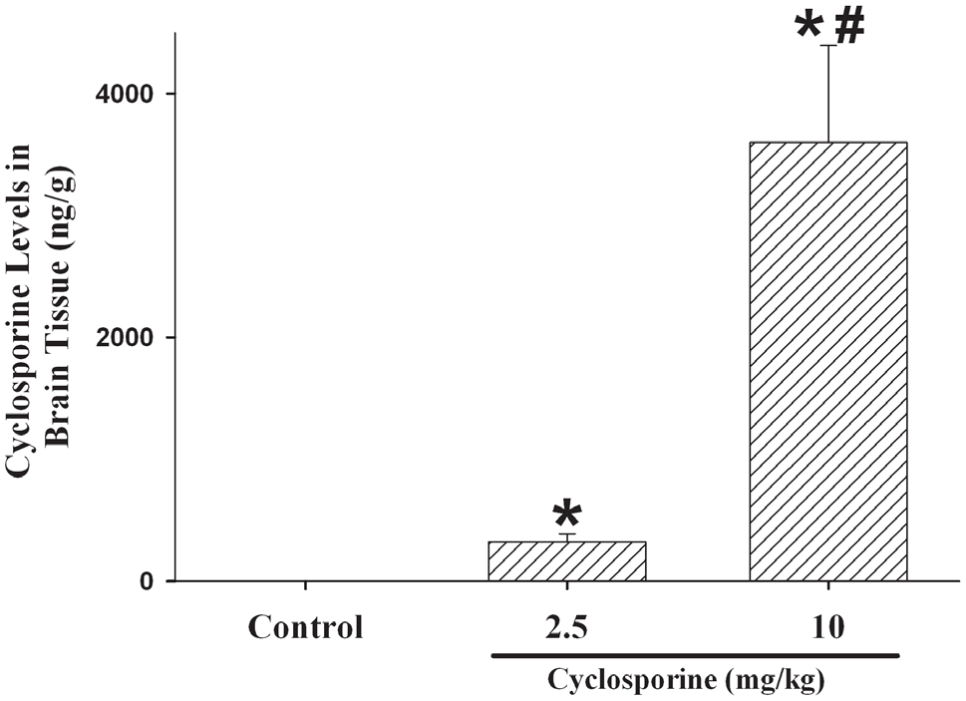
Cerebral cortical levels of cyclosporine in piglets after hypoxia-reoxygenation. Piglets received either saline (control) or cyclosporine 2.5 (CSA 2.5) or 10 mg/kg (CSA 10) 5 min after reoxygenation (n = 8 each). *p<0.05 vs. H-R controls, ^#^p<0.05 vs. CSA 2.5 group (1-way ANOVA).

### Effects of Cyclosporine on Cortical Glutathione and Lactate Levels

In the cerebral cortical tissue following H-R, there was an increase in GSSG levels in the H-R control group (vs. sham-operated piglets, p<0.05) (Table 2). Cortical GSSG levels were significantly reduced in piglets treated with 10 mg/kg cyclosporine compared to H-R controls and were similar to levels of sham-operated piglets. Total GSH levels were not different among groups (Table 2). These changes in GSSG and GSH levels resulted in a significant increase in glutathione redox (GSSG:GSH) ratio in the H-R controls, but not in cyclosporine-treated groups (Table 2).

The H-R control piglets had significantly higher cerebral cortical lactate levels than that of sham-operated piglets at the end of experiment (Fig. 4). Treating the animal with 10 mg/kg cyclosporine significantly reduced the cortical lactate level compared to H-R controls (Fig. 4).

Overall, the cumulative cortical H_2_O_2_ concentration was significantly correlated with cytosol cytochrome-c level (r = 0.45, p<0.05) and cortical GSSG (r = 0.51, p<0.01), but not cortical GSH level or GSSG:GSH redox ratio. The cumulative cortical H_2_O_2_ concentration also correlated positively cortical lactate levels (r = 0.77, p<0.01). No significant correlation was found between cortical H_2_O_2_ concentration and simultaneous CABF, heart rate or MAP, GSH and lactate levels (data not shown).

### Cyclosporine A Levels in the Brain in Newborn Piglets

After a single, bolus, intravenous injection of cyclosporine A which was administered at 5 min of reoxygenation, cyclosporine was detected in appreciable concentrations in the cerebral cortical tissue in these newborn H-R piglets at 4 h of reoxygenation (p<0.05 vs. H-R controls). The tissue levels were significantly higher in the piglets treated with 10 than with 2.5 mg/kg (Fig. 5).

## Discussion

This is the first study to demonstrate that post-resuscitation cyclosporine treatment attenuates (1) cortical H_2_O_2_ concentration and oxidative stress, (2) cortical lactate and cytosol cytochrome-c levels in newborn piglets during reoxygenation after a severe hypoxic insult, with no associated changes in carotid hemodynamics. These findings support the therapeutic potential of cyclosporine as a neuroprotective agent of cyclosporine in neonatal asphyxia with its attenuation of H-R induced cerebral damage.

Although several studies have evaluated the effectiveness of cyclosporine treatment following hypoxic-ischemic injury to the immature brain [8], [13], [14], [15], none of these studies examined the effectiveness of cyclosporine treatment on ROS generation. Using specific electrochemical H_2_O_2_ sensors, we were able to directly monitor the change in cortical H_2_O_2_ production continuously during H-R. The cortical H_2_O_2_ concentration remained near baseline during hypoxia. Upon resuscitation, the cortical H_2_O_2_ concentration of H-R control piglets increased gradually during the early period of reoxygenation and then became markedly elevated at 2 h post-reoxygenation. Interestingly, this observation is different from previous reports of a rapid surge of ROS, particularly nitric oxide, immediately after reoxygenation/reperfusion [23], [24]. We are not certain about the etiology but speculate that the slow rise of cortical H_2_O_2_ concentration could be related to the competitive reactions of nitric oxide and superoxide dismutase for superoxide anions. Beckman et al showed a three-fold difference in rate constants between the nitric oxide reaction and enzymatic dismutation with superoxide anions (6.3 vs. 2.3×10^−6^ M/s, respectively) [25]. Further, it has been recently shown that reduction of nitric oxide may lead to enhanced formation of H_2_O_2_ [26].

Post-resuscitation cyclosporine treatment significantly attenuated the *in vivo* rise in H_2_O_2_ similar to levels observed in the sham-operated piglets. In regard to tissue markers of cortical oxidative stress, we observed that increased GSSG levels and GSSG/GSH ratio in the cortex of H-R control piglets following reoxygenation were significantly attenuated by cyclosporine treatment. Further, a positive correlation was found between the cumulative cortical H_2_O_2_ and GSSG concentrations. A significant reduction in ROS has been reported previously in isolated mitochondria from ischemic cells following treatment by cyclosporine or its non-immunosuppressive analog [27], [28], [29]. Furthermore, cyclosporine has been shown to inhibit H_2_O_2_ generation in isolated brain and liver mitochondria exposed to excess calcium [30]. Mitochondria are a major source of ROS generation during reoxygenation/reperfusion. ROS produced by mitochondria may lead to the release of calcium from the endoplasmic reticulum, resulting in mitochondrial calcium over-loading, MPTP opening and further ROS production [7], [30], [31], [32], [33]. Cyclosporine has been shown in *in vitro* experiments to maintain mitochondrial homeostasis by binding to cyclophilin D and preventing MPTP opening. Interestingly, the opening of MPTP itself may induce ROS production at complex I of the respiratory chain [34]. It has been previously reported that MPTP opening may enhance cytochrome-c dislocation from the mitochondrial inner membrane and its subsequent release into the cytosol [6], [7], [8]. Therefore, we suggest that observed reduction in H_2_O_2_ production following cyclosporine treatment in the present study may be related to cyclosporine’s inhibitory effect on MPTP opening. This speculation is supported by our observations on cytosol cytochrome-c levels and the significant correlation between cortical H_2_O_2_ concentrations and cytochrome-c levels. However, further research with direct measurement of MPTP activation is needed to verify these speculations.

Despite preserved regional blood flow and brain tissue oxygen tension, increases in brain extracellular lactate have been reported in patients with head injury [35], [36]. It was suggested that the increase in lactate concentration may be due to inefficiency of mitochondrial oxidative metabolism. The significant increase in cortical lactate level in H-R control piglets was reduced by cyclosporine, in the absence of carotid hemodynamic effects. A direct measurement of cerebral perfusion will help understand if cerebral hemodynamics was affected by cyclosporine treatment. Similar reductions in brain lactate levels by cyclosporine treatment after hypoxia-ischemia have also been reported recently in newborn rats [13]. These results indicate that cyclosporine may attenuate mitochondrial metabolic impairment in newborn subjects. Interestingly, we did not observe any associative improvement in carotid hemodynamics. However, cyclosporine or its non-immunosuppressive analogs have been shown recently to improve cerebral blood flow in rats after cortical spreading depression [37]. These differences may be related to methodology for blood flow assessment, cyclosporine dosage, and the animal model used. Furthermore, an organ-specific effect of cyclosporine on brain lactate may be possible as plasma lactate levels were similar in all H-R groups.

Interestingly, we detected appreciable levels of cyclosporine A in the cortical tissue of newborn piglets after H-R (Fig. 5). Although cyclosporine is typically limited from reaching the brain, during H-R the blood-brain-barrier has been shown to have increased permeability [38]. Okonkwo et al observed similar cyclosporine brain concentrations in rats following traumatic brain injury with intravenous cyclosporine at 20 mg/kg and intrathecal cyclosporine at 10 mg/kg [39]. Indeed, Pacho-Lopez et al, in a study of adult rats after an intra-peritoneal injection of cyclosporine (20 mg/kg) [40], demonstrated progressive accumulation of cyclosporine levels in the brain tissue at 120 and 240 min after administration whereas the blood levels decreased. While we do not know if the presence of cyclosporine in the cerebral cortical tissue is related to the immaturity or H-R damage or species difference of blood-brain-barrier, the possibility of a “direct” neuroprotective effect of cyclosporine needs to be further investigated.

There are several limitations to consider in addition to those of animal experimentation. Firstly, we did not directly observe *in vivo* MPTP activation in the brains of newborn piglets. Instead we used indirect markers of oxidative stress to identify cortical damage due to H-R. Secondly, during H-R tremendous amount of nitric oxide and nitrogen radicals like peroxynitrite are generated and also cause mitochondrial damage. Studies on nitric oxide or nitric oxide metabolites level at each time point will further our understanding on the mitochondrial function and MPTP activation in the nitric oxide pathway. Lastly, in 6 h of experimentation, it is uncertain if the attenuation of oxidative stress will translate into improved functional or neurologic recovery in prolonged observing or long-term period [41].

Taken together, our findings support the likelihood that cyclosporine may exert its neuroprotective effects by preventing MPTP opening, with reduced oxidative stress and preserved energy homeostasis, in a newborn piglet model of H-R. Further research is warranted to confirm if the post-resuscitation administration of cyclosporine provides neuroprotection against oxidative stress-related injury in asphyxiated neonates.
